# A novel pancoronavirus RT-PCR assay: frequent detection of human coronavirus NL63 in children hospitalized with respiratory tract infections in Belgium

**DOI:** 10.1186/1471-2334-5-6

**Published:** 2005-02-01

**Authors:** Elien Moës, Leen Vijgen, Els Keyaerts, Kalina Zlateva, Sandra Li, Piet Maes, Krzysztof Pyrc, Ben Berkhout, Lia van der Hoek, Marc Van Ranst

**Affiliations:** 1 Laboratory of Clinical & Epidemiological Virology, Department of Microbiology & Immunology, Rega Institute for Medical Research, University of Leuven, Belgium; 2 Department of Human Retrovirology, Academic Medical Center, University of Amsterdam, The Netherlands

## Abstract

**Background:**

Four human coronaviruses are currently known to infect the respiratory tract: human coronaviruses OC43 (HCoV-OC43) and 229E (HCoV-229E), SARS associated coronavirus (SARS-CoV) and the recently identified human coronavirus NL63 (HCoV-NL63). In this study we explored the incidence of HCoV-NL63 infection in children diagnosed with respiratory tract infections in Belgium.

**Methods:**

Samples from children hospitalized with respiratory diseases during the winter seasons of 2003 and 2004 were evaluated for the presence of HCoV-NL63 using a optimized pancoronavirus RT-PCR assay.

**Results:**

Seven HCoV-NL63 positive samples were identified, six were collected during January/February 2003 and one at the end of February 2004.

**Conclusions:**

Our results support the notation that HCoV-NL63 can cause serious respiratory symptoms in children. Sequence analysis of the S gene showed that our isolates could be classified into two subtypes corresponding to the two prototype HCoV-NL63 sequences isolated in The Netherlands in 1988 and 2003, indicating that these two subtypes may currently be cocirculating.

## Background

Coronaviruses are large, enveloped, positive stranded RNA-viruses [1]. The viral RNA genome is 27–32 kb in size, capped, polyadenylated and encapsidated in a helical nucleocapsid. The envelope is studded with long, petal-shaped spikes, giving the virus particle a characteristic crown-like appearance. Three distinct groups of coronaviruses have been described based on serological affinity and genome sequence. Coronaviruses can infect humans and a variety of domestic animals and can cause highly prevalent diseases such as respiratory, enteric, cardiovascular and neurologic disorders [2,3].

Until recently only three human coronaviruses were thoroughly studied. Human coronavirus OC43 (HCoV-OC43; group 2) and human coronavirus 229E (HCoV-229E; group 1) were identified in the 1960s. They are responsible for 10–30% of all common colds, and infections occur mainly during winter and early spring [4-7]. A third novel human coronavirus, SARS-CoV, was identified as the causal agent during the 2002–2003 outbreak of severe acute respiratory syndrome (SARS) [8-10]. Phylogenetic analysis showed that the SARS-CoV does not closely resemble any of the three previously known groups of coronaviruses, and therefore a tentative fourth group of coronaviruses was suggested [11,12]. However, an early split-off of the SARS-CoV from the coronavirus group 2 lineage has also been suggested [13,14].

A new human coronavirus associated with respiratory illness, HCoV-NL63, was recently identified by a research team in The Netherlands [15]. The virus was isolated in January 2003 from a nasopharyngeal aspirate of a 7-month-old child suffering from bronchiolitis, conjunctivitis and fever. Screening of specimens from patients with respiratory symptoms identified seven additional HCoV-NL63 infected individuals, both children and adults, between December 2002 and February 2003. The complete viral genome sequence was determined. The characteristic genome organisation of coronaviruses can be observed: the 5' two-third of the genome contains two large open reading frames (ORF), ORF1a and ORF1b. In the 3' part of the genome, genes encoding four structural proteins are found: spike (S), envelope (E), membrane (M), and nucleocapsid (N). The hemagglutinin-esterase (HE) gene, characteristic for group 2 coronaviruses, is not present in HCoV-NL63. Sequence analysis demonstrated that HCoV-NL63 shares 65% sequence identity with HCoV-229E. Phylogenetic analysis confirmed that HCoV-NL63 is a new group 1 coronavirus, most closely related to HCoV-229E and porcine epidemic diarrhea virus (PEDV) [15]. Shortly after van der Hoek and colleagues published their discovery of the new human coronavirus HCoV-NL63, a second research group described the characterization of essentially the same virus [16]. The virus was isolated from a nose swab sample collected from an 8-month-old child suffering from pneumonia in The Netherlands in April 1988. Real-time RT-PCR assays were designed for screening of respiratory tract samples. Four additional HCoV-NL63 positive samples, from children aged 3 months to 10 years, were detected between November 2000 and January 2001.

HCoV-NL63 can be considered as a new important cause of respiratory illnesses and two different subtypes might be currently cocirculating in the human population [15]. In this study we wanted to explore the incidence of HCoV-NL63 infection in children diagnosed with respiratory tract infections in Belgium.

## Methods

### Isolates and patients

**Figure 1:**
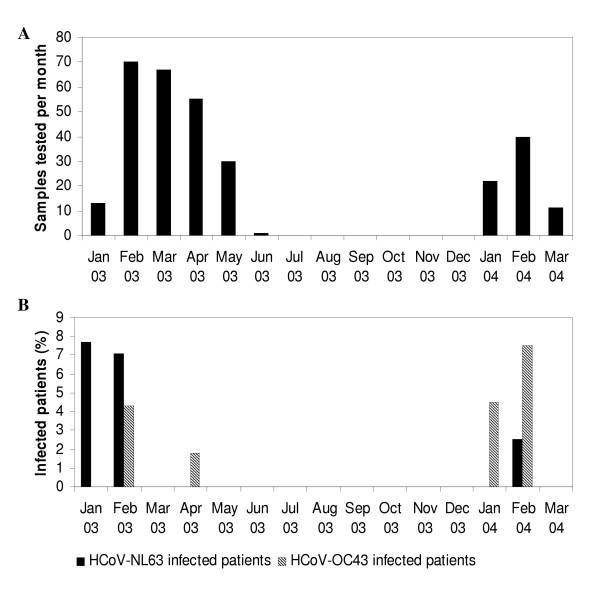
Detection of HCoV-NL63 and HCoV-OC43 in samples from patients suffering from severe respiratory symptoms. (A) Number of samples tested per month. (B) Patients infected with HCoV-NL63 and HCoV-OC43. A single HCoV-229E positive sample was isolated in April 2003 (not shown).

We studied 309 isolates from 279 patients with severe respiratory symptoms collected from January 2003 until March 2004 at the University Hospital in Leuven, Belgium. These isolates originated from bronchoalveolar lavages, pharyngeal swabs, nasopharyngeal aspirates, and sputum samples. Routine diagnostic testing was performed for respiratory syncytial virus (RSV), influenza virus, parainfluenza virus and adenovirus. No prior amplification by cell culture was performed. The results of diagnostic tests for RSV were negative for 244 isolates, while 65 isolates were positive for RSV. Patients ranged in age from 1 month to 16 years, with a mean age of 2 years. The temporal distribution of the isolates corresponded to the yearly RSV epidemic period: 236 samples were collected from January to June 2003 and 73 samples were recovered during the first trimester of 2004 (Figure 1A).

### Pancoronavirus RT-PCR assay

**Figure 2:**
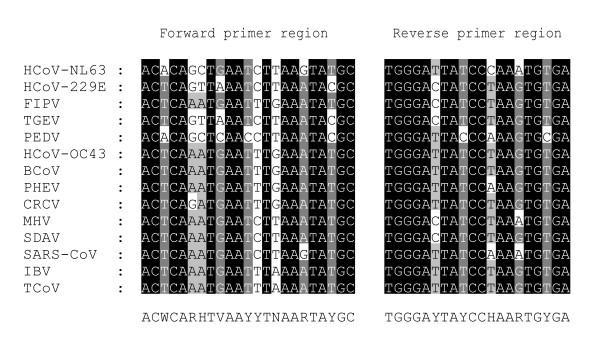
Selection of primers for the novel pancoronavirus RT-PCR. Shown is the alignment of 14 coronaviral sequences of a conserved region of the polymerase gene. The forward (Cor-FW) and reverse (Cor-RV) primer sequences are shown at the bottom (Y = C/T, W = A/T, V = A/C/G, R = A/G, H = A/T/C, N = A/C/T/G). The coordinates of Cor-FW and Cor-RV are 14017 and 14248, respectively, in the HCoV-NL63 complete genome sequence. The 14 coronavirus sequences used here are available from GenBank under the following accession numbers: HCoV-NL63, AY567487; HCoV-229E, AF304460; infectious bronchitis virus (IBV), Z30541; SARS-CoV, AY313906; HCoV-OC43, AY391777; PEDV, AF353511; bovine coronavirus (BCoV), AF391541; transmissible gastroenteritis virus, AF304460; MHV, X51939; PHEV, AF124988; sialodacryoadenitis virus (SDAV), AF124990; turkey coronavirus (TCoV), AF124991; canine respiratory coronavirus (CRCV), AY150273; feline infectious peritonitis virus (FIPV), AF124987.

RNA was extracted from the collected specimens by using the QIAamp Viral RNA Mini kit (QIAGEN, Westburg, The Netherlands) according to instructions of the manufacturer. Screening of the samples was performed by amplifying a 251 bp fragment of the polymerase gene using the following primer set: Cor-FW (5'-ACWCARHTVAAYYTNAARTAYGC-3') and Cor-RV (5'-TCRCAYTTDGGRTARTCCCA-3') (Figure 2). These one-step RT-PCR assays (OneStep RT-PCR kit; QIAGEN) were undertaken in a 50 μl reaction volume containing 10 μL RNA-extract, 10 μl 5x QIAGEN OneStep RT-PCR Buffer, 2 μl dNTP mix (final concentration of 400 μM of each dNTP), 1.8 μl QIAGEN OneStep RT-PCR Enzyme Mix (a combination of Omniscript and Sensiscript reverse transcriptase and HotStarTaq DNA polymerase), 4 μM of each primer, and RNase-free water to 50 μl. The reaction was carried out with an initial reverse transcription step at 50°C for 30 min, followed by PCR activation at 95°C for 15 min, 50 cycles of amplification (30 sec at 94°C; 30 sec at 48°C; 1 min at 72°C), and a final extension step at 72°C for 10 min in a GeneAmp PCR system 9600 thermal cycler (Applied Biosystems, Foster City, CA, USA). PCR-products were run on a polyacrylamide gel, stained with ethidium bromide, and visualized under UV-light.

### RT-PCR assays for HCoV-NL63

Samples that were found positive for HCoV-NL63 were confirmed using one-step RT-PCR assays, which amplified four different regions of the HCoV-NL63 genome. Amplification of a 314-bp gene fragment in the nucleocapsid region was performed with two specific HCoV-NL63 primers: N5-PCR1 (5'-CTGTTACTTTGGCTTTAAAGAACTTAGG-3', nt 26695-nt 26721) and N3-PCR1 (5'-CTCACTATCAAAGAATAACGCAGCCTG-3', nt 26982-nt 27008). Secondly a 237-bp fragment in ORF1b was amplified using the primers repSZ-1 and repSZ-3 described by van der Hoek and colleagues [15]. A third RT-PCR assay was carried out on the HCoV-NL63 positive samples amplifying a 839-bp fragment with ORF1a specific primers: SS5852-5P and P4G1M-5-3P [15]. These one-step RT-PCR assays were performed essentially as described above. They were carried out using 5 μL RNA-extract and 0.6 μM of each primer. Only 45 cycles of amplification were run and annealing temperature was set at 50°C. Furthermore a 663 bp fragment of the spike gene was amplified using a RT-nested PCR. The outer primer set SINL5 (5'-GAGTTTGATTAAGAGTGGTAGGTTG-3', nt 20391-nt 20415) and SINL3 (5'-AACAGTGTAGTTAACTACACGG-3', nt 21068-nt 21089) were used in a one-step RT-PCR, performed as described above, using 10 μl of RNA-extract and an annealing temperature of 48°C. A nested PCR was carried out with the inner primer set SINL5n (5'-GGTTGTTGTTACGCAATAATGGTCGT-3', nt 20411-nt 20436) and SINL3n (5'-ACACGGCCATTATGTGTGGTGAC-3', nt 21051-nt 21073). The nested reaction mix was composed of 1 unit Taq polymerase, 1 μl of a 25 mM dNTP-mix, 10 μl 5X buffer C (PCR Optimizer Kit, Invitrogen, The Netherlands), and 30 pmol of forward and reverse primer in a 50 μl reaction volume. As template 10 μl of the outer PCR product was added. The cycling conditions were as follows: an initial denaturation at 94°C for 5 min, followed by 40 cycles of amplification (45 sec at 94°C, 45 sec at 54°C, 1 min at 72°C), and a final extension of 5 min at 72°C. PCR-products were run on a polyacrylamide gel, stained with ethidium bromide, and visualized under UV-light. The amplicons were purified using the QIAquick PCR purification kit (QIAGEN) and sequenced with the respective primer pairs using the ABI PRISM BigDye Terminator Cycle Sequencing Reaction kit (version 3.1) on an ABI PRISM 3100 DNA sequencer (Applied Biosystems) according to the manufacturer's instructions. Positive and negative controls were included in each PCR experiment. The HCoV-NL63 positive control was RNA isolated from a HCoV-NL63 culture.

### Sequence analysis and phylogenetic analysis of the amplicons

Chromatogram sequencing files were inspected with Chromas 2.2 (Technelysium Pty Ltd, Helensvale, Australia), and contigs were prepared using SeqMan II (DNASTAR, Madison, WI, USA). The obtained consensus sequences were compared with the prototype HCoV-NL63 sequences available in GenBank database release 142.0 using BLAST analysis (NCBI BLAST server). Multiple sequence alignments were prepared using CLUSTAL X version 1.82 [26], and manually edited in the GeneDoc Alignment editor [27]. Phylogenetic analysis was conducted using MEGA version 2.1 [28].

### Nucleotide sequence accession numbers

The sequences determined in this study have been deposited in the GenBank sequence database under accession numbers AY758276 to AY758301.

### RT-PCR assays for HCoV-OC43 and HCoV-229E

Our collection of samples was also screened using the pancoronavirus RT-PCR assay for the presence of HCoV-OC43 and HCoV-229E. Positive results were confirmed by one-step RT-PCR using HCoV-OC43 and HCoV-229E specific primer pairs located in the membrane glycoprotein region (OC43-FW: 5'-GGCTTATGTGGCCCCTTACT-3', nt 28580-nt 28599; OC43-RV: 5'-GGCAAATCTGCCCAAGAATA-3', nt 28894-nt 28913; 229E-FW: 5'-TGGCCCCATTAAAAATGTGT-3', nt 24902-nt 24921; 229E-RV: 5'-CCTGAACACCTGAAGCAAT-3', nt 25456-nt 25475) [18]. One-step RT-PCR and sequence analysis were performed essentially as described above. Annealing conditions during the RT-PCR assay were modified: the annealing temperature was set at 55°C.

## Results

### Pancoronavirus RT-PCR assay

**Figure 3:**
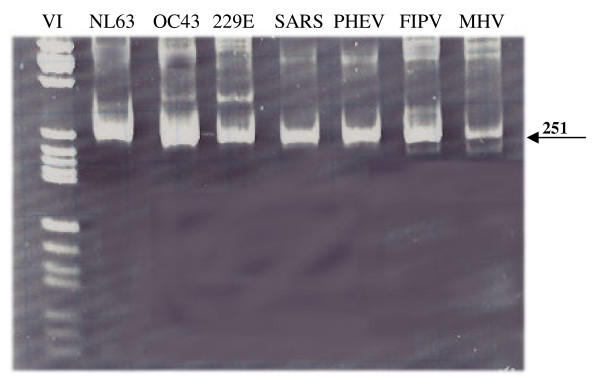
Gel electrophoresis after pancoronavirus RT-PCR assay. The indicated band of 251 bp corresponds with the expected amplicon size. As a marker Molecular Weight Marker VI was used (Boehringer Mannheim, Germany).

A pancoronavirus RT-PCR assay is a usefull tool to test for all coronaviruses in a clinical sample. Besides quick screening for several pathogens in one assay, it supplies the possibility to identify previously unknown coronaviruses. The consensus RT-PCR assay as described by Stephensen et al., designed to amplify all known coronaviruses, is not able to detect HCoV-NL63 because of several mismatches with the primer sequences [15,17]. We modified these consensus primers based on an alignment of the HCoV-NL63 prototype sequence and 13 other coronavirus sequences (Figure 2). To determine whether the newly designed pancoronavirus RT-PCR assay efficiently amplifies a broad range of coronaviruses the RT-PCR assay was tested on cell culture supernatant of the four known human coronaviruses and three animal coronaviruses: HCoV-NL63, HCoV-OC43, HCoV-229E, SARS-CoV, feline infectious peritonitis virus (FIPV), porcine hemagglutinating encephalomyelitis virus (PHEV), and murine hepatitis virus (MHV). Amplification of the expected 251 bp region was observed for all tested coronaviruses (Figure 3). The sensitivity of the pancoronavirus RT-PCR assay was assessed by testing tenfold dilutions of HCoV-NL63 and HCoV-OC43 RNA. While 50 copies of HCoV-OC43 RNA copies per μl nasopharyngeal aspirate could be detected, the sensitivity for HCoV-NL63 was a bit lower i.e. 5 × 10^3 ^RNA copies per μl nasopharyngeal aspirate.

### Detection of HCoV-NL63 in clinical specimens

The pancoronavirus RT-PCR assay was used for screening of specimens from hospitalized patients with respiratory symptoms collected between January 2003 and March 2004. Samples, from which a 251 bp fragment could be amplified, were further identified by sequencing using the pancoronavirus primers. We studied 309 specimens with a temporal distribution that corresponded with the yearly RSV epidemic period (Figure 1A). A total of 244 samples were found negative for RSV by diagnostic testing. The 279 patients in this study comprised of 211 patients aged <2 years (75.6%), 68 aged 2–16 years (24.4%). We detected HCoV-NL63 in 7 samples (2.3%). One positive sample was collected at the end of January 2003 and coinfection with RSV type B was present. Five of the positive samples were collected within a ten-day period at the end of February 2003, and one positive sample was collected at the end of February 2004, which showed coinfection with adenovirus and parainfluenza virus (Figure 1B, Table 1). The seven positive samples were obtained from one patient aged 1 month, four patients of 1 year, one patient of 2 years, and one patient of 16 years. The patient files showed that all subjects suffered from respiratory tract illness and some had underlying disease (Table 1).

**Table 1:** Patients hospitalized with respiratory tract illness associated with HCoV-NL63 infection

Patient nr.	Age	Sex	Symptoms	Underlying disease	Specimen	Sample date
1153^a^	1 year	male	URTI: fever, coughing, wheezing, rhinitis, diarrhoea	none	NPA	27 Jan 2003
33545	16 years	male	LRTI: fever, coughing, respiratory distress, pharyngitis	Smith-Lemli-Opitz syndrome	NPA	14 Feb 2003
21596	1 year	female	LRTI: fever, coughing, respiratory distress	Vater syndrome, epilepsy	NPA	20 Feb 2003
53887	1 month	female	URTI: fever, rhinitis, two siblings have URTI	none	NPA	20 Feb 2003
40001	1 year	male	LRTI: respiratory distress, cardiac arrest, rotavirus-positive diarrhoea	epilepsy	NPA	21 Feb 2003
64880	2 years	male	URTI: fever, coughing, wheezing	neurofibromatosis	NPA	24 Feb 2003
70688^b^	1 year	female	LRTI: pneumonia, fever, cyanosis, diarrhoea	none	PS	25 Feb 2004

^a^positive for RSV type B; ^b^positive for adenovirus and parainfluenza virus

LRTI, lower respiratory tract illness; URTI, upper respiratory tract illness; PS, pharyngeal swab; NPA, nasopharyngeal aspirate

**Figure 4:**
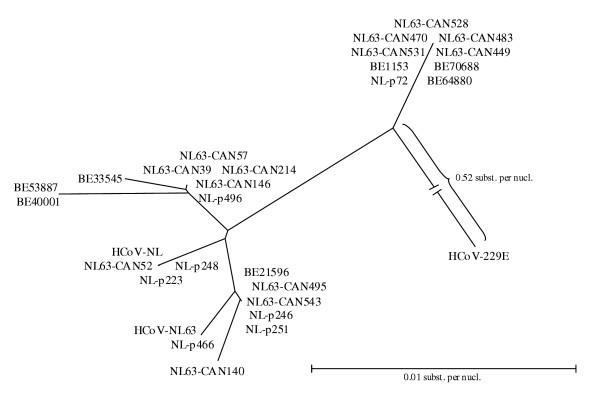
Phylogenetic analysis of the partial ORF1a nucleotide sequences. Accession numbers: HCoV-NL63, AY567487; HCoV-NL, AY518894; HCoV-229E, AF304460; NL-p466, AY567488; NL-p246, AY567489; NL-p251, AY567490; NL-p496, AY567491; NL-p223; AY567492; NL-p248, AY567493; NL-p72, AY567494; CAN39, AY675541; CAN52, AY675542; CAN57, AY675543; CAN140, AY675544; CAN146, AY675545; CAN214, AY675546; CAN449, AY675547; CAN470, AY675548; CAN483, AY675549; CAN495, AY675550; CAN528, AY675551; CAN531, AY675552; CAN543, AY675553.

The seven HCoV-NL63 positive respiratory samples were confirmed by alternative RT-PCR assays. Amplification of a fragment of the nucleocapside gene and ORF1b was carried out. Sequence analysis of the N gene fragments and the ORF1b fragments showed 98–100% similarity to the prototype HCoV-NL63 sequences available in the GenBank database (AY567487, AY518894). A third one-step RT-PCR was carried out for each positive sample to amplify part of the ORF1a gene. Sequence analysis of the ORF1a PCR-products revealed 99% sequence identity with both HCoV-NL63 prototype sequences available in GenBank. A neighbor-joining phylogenetic tree was constructed based on an alignment of the ORF1a nucleotide sequences from the HCoV-NL63 positive samples and the available HCoV-NL63 sequences in GenBank. HCoV-229E was used as an outgroup. The dendrogram shows that all HCoV-NL63 sequences cluster together, but two subclusters can be observed (Figure 4).

**Figure 5:**
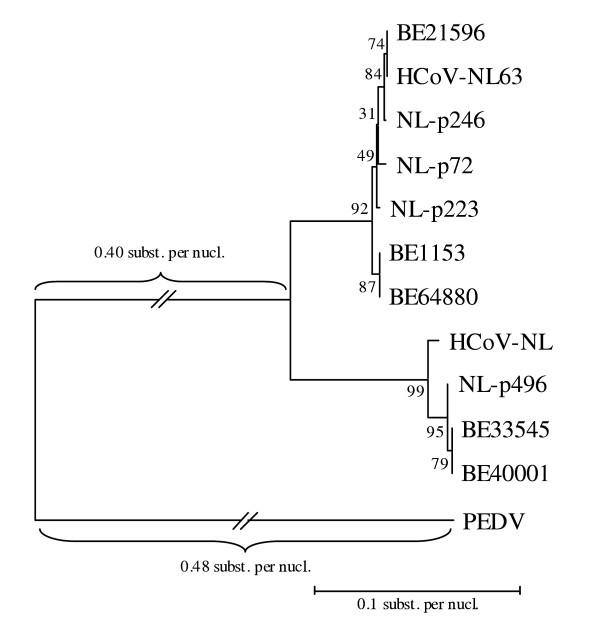
Phylogenetic analysis of the partial S gene nucleotide sequences based on an alignment of the Belgian spike sequences, spike sequences from the positive samples identified in The Netherlands, and the prototype HCoV-NL63 sequences available in GenBank. Accession numbers: HCoV-NL63, AY567487; HCoV-NL, AY518894. PEDV was used as an outgroup.

Inspection of the two full genome HCoV-NL63 sequences available in GenBank demonstrates that especially the aminoterminal region of the Spike protein can be very divergent. Therefore we decided to amplify this region to investigate the variability of these region in our patients. An RT-nested PCR assay was used to amplify part of the S gene. These partial spike sequences showed 98% similarity with the HCoV-NL63 prototype strains. An alignment of the S gene sequences from the Belgian samples, partial spike sequences from the positive samples identified in The Netherlands (data not shown), and the prototype HCoV-NL63 sequences, was used to constitute a neighbor-joining phylogenetic tree. The neighbor-joining tree was evaluated by 500 bootstrap pseudoreplicates. Two clusters can again be observed (Figure 5).

### Detection of HCoV-OC43 and HCoV-229E

Screening of our sample collection for the presence of HCoV-OC43 and HCoV-229E was also performed. We detected HCoV-OC43 in 7 of 309 samples (2.3%) and HCoV-229E in one sample (0.3%). The seven HCoV-OC43 positive samples were collected during the winter and early spring of 2003 and 2004. The sample in which we detected HCoV-229E was collected in April 2003. The positive samples were confirmed by RT-PCR using specific HCoV-OC43 and HCoV-229E primer pairs that amplify part of the M gene [18]. The HCoV-OC43 and HCoV-229E partial membrane sequences of the contemporary Belgian strains showed 97–99% similarity with the HCoV-OC43 and HCoV-229E prototype sequences in GenBank.

## Discussion

RSV, influenza viruses, adenoviruses, and parainfluenzaviruses are probably the most important viral agents of severe respiratory diseases. However, a substantial part of respiratory tract infections can not be attributed to any known pathogen. Underlying conditions and immunosuppression largely determine the impact of respiratory viruses on individuals [19]. The common cold viruses HCoV-OC43 and HCoV-229E have also been associated with more severe lower respiratory tract conditions in infants and immunocompromised patients [20-23]. The clinical symptoms associated with HCoV-NL63 infections still need to be determined, but there are some indications that HCoV-NL63 can cause severe respiratory illnesses in children and immunocompromised adults [15,16]. We detected HCoV-NL63, using a pancoronavirus RT-PCR, in patients suffering from relatively severe respiratory diseases necessitating hospitalization. These positive samples were collected from children aged 1 month to 16 years. Two patients suffered from severe underlying disease: one patient suffered from Smith-Lemli-Opitz syndrome, a rare autosomal recessive disorder due to a primary enzymatic defect in the cholesterol metabolism. A second patient was diagnosed with VATER, a syndrome characterized by the sporadic association of specific birth defects or abnormalities such as vertebrae and vascular anomalies, anal atresia, trachea and esophagus problems and renal anomalities. All HCoV-NL63 infected patients established a complete recovery from their respiratory symptoms. One-step RT-PCR assays were used to detect and confirm these positive samples.

Results from epidemiological surveys conducted in the 1970's have led to the conclusion that human coronaviruses are distributed worldwide and circulate during seasonal outbreaks [22]. Our results indicate that HCoV-NL63 is the causal agent in a significant portion of respiratory diseases of unknown etiology. We detected HCoV-NL63 in respiratory samples collected in February 2003, with a frequency of 7.1%, and during February 2004, with a frequency of 2.5%. These results seem to support the tendency of human coronaviruses to circulate mainly during the winter season [7,24]. However, in this study, sampling was only performed from January to May during the yearly RSV epidemic period, while no samples from the summer and autumn months were screened. The first publication on HCoV-NL63 showed that the virus circulated in Amsterdam during the winter months of 2002/2003 [15]. More recently another set of Amsterdam samples was screened, obtained during the winter of 2001/2002 and 2003/2004. HCoV-NL63 was found in one trachea sample obtained in February 2002, and in two oropharyngeal aspirates from December 2003 and January 2004, respectively (data not shown). Combined with the data that we present here from Belgium, these findings confirm that HCoV-NL63 reappears each winter season similar to the previously known respiratory viruses. Recently, research teams from Australia, Japan and Canada, have submitted partial HCoV-NL63 sequences to the GenBank database (AY600442-AY600446, AY662694-AY662698, AY675541-AY675553). This indicates that this newly discovered human coronavirus has a worldwide distribution.

Sequence analysis of the highly conserved nucleocapsid region showed that the Belgian isolates are similar to the two prototype HCoV-NL63 complete genome sequences in GenBank isolated in the Netherlands in 1988 and 2003. Furthermore, phylogenetic analysis of part of the ORF 1a region of our patients showed the same subclusters of HCoV-NL63 that were described previously [15] (Figure 4). This finding supports the suggestion that several HCoV-NL63 subtypes with distinct molecular markers are cocirculating, also in Belgium. A large insert in the 5' part of the S gene of HCoV-NL63 compared with HCoV-229E has been described [15,16]. Both HCoV-NL63 complete genome sequences show only 89% sequence identity in this spike insert region, which implies that there are at least two different HCoV-NL63 subtypes. Sequence analysis of this spike insert region revealed that our samples show similarity to both prototype HCoV-NL63 subtypes, which was confirmed by phylogenetic analysis. The partial S gene sequences cluster together with the two prototype HCoV-NL63 sequences in two different groups (Figure 5). This confirms that the HCoV-NL63 subtypes first isolated in 1988 and 2003 are cocirculating. When analysing the dendrograms based on ORF1a and S gene sequences, a discordance in the clustering pattern of some HCoV-NL63 isolates (e.g. HCoV-NL and NL-p223) can be observed, suggesting a possible recombination event. Further research of complete genome sequences of these isolates is required. Drawing conclusions based on phylogenetic analysis of one single gene therefore requires caution as the true phylogeny can only be demonstrated by analysing complete genome sequences.

Screening of our sample collection for the presence of HCoV-OC43 and HCoV-229E revealed seven HCoV-OC43 positive samples and only one HCoV-229E positive sample. All positive samples were isolated during winter and early spring, which is concordant with the results of previous epidemiological studies. HCoV-OC43 infected samples were mainly identified during February 2003 and February 2004 (Figure 1B). These data show that the epidemic seasons of HCoV-OC43 and HCoV-NL63 coincide. The positive samples were collected from children aged 1 to 12 years, whom all suffered from respiratory symptoms. The very low detection rate of HCoV-229E compared with the frequent detection of HCoV-NL63, might imply that HCoV-NL63, closely related to HCoV-229E, is currently more important as a causal agent of respiratory diseases. At the moment, there are no data concerning cross-neutralization between HCoV-229E and HCoV-NL63. In theory, such cross-neutralization might be possible, since both viruses are relatively closely related species belonging to coronavirus group 1. Antigenic cross-reactivity has already been demonstrated between SARS-CoV and group 1 coronaviruses TGEV, FIPV and CCoV [25].

The development of a pancoronavirus RT-PCR assay using a primer set that matches all known coronaviruses might be useful for the identification of new coronaviruses. This pancoronavirus RT-PCR-assay can also be used as a diagnostic tool to detect any of the four currently known human coronaviruses in clinical samples.

## Conclusions

Human coronavirus NL63 is a new important respiratory pathogen that can cause severe respiratory infections in children. Sequence analysis of the HCoV-NL63 isolates detected in our study demonstrates that our Belgian isolates can be classified into two subtypes corresponding to the two prototype HCoV-NL63 sequences isolated in The Netherlands in 1988 and 2003. Our findings indicate that these two subtypes may currently be cocirculating.

## Competing interests

The author(s) declare that they have no competing interests.

## Author's contributions

EM conceived of the study and designed it together with LV, EK, and MVR. EM developed the pancoronavirus RT-PCR and performed the RT-PCR and sequencing reactions. EM and LV drafted the manuscript. KZ assembled the respiratory samples. SL performed the RT-PCR sensitivity assays. PM was responsible for the graphical support of the manuscript. LVDH, KP and BB developed the HCoV-NL63 RT-PCRs and helped with the design of the study and the writing of the manuscript. All authors contributed to the final version of the manuscript, read and approved it.

## Pre-publication history

The pre-publication history for this paper can be accessed here:


